# An Oily Fish Diet Improves Subclinical Inflammation in People at High Cardiovascular Risk: A Randomized Controlled Study

**DOI:** 10.3390/molecules26113369

**Published:** 2021-06-02

**Authors:** Giuseppina Costabile, Giuseppe Della Pepa, Claudia Vetrani, Paola Vitaglione, Ettore Griffo, Rosalba Giacco, Marilena Vitale, Dominic Salamone, Angela Albarosa Rivellese, Giovanni Annuzzi, Lutgarda Bozzetto

**Affiliations:** 1 Department of Clinical Medicine and Surgery, Federico II University, 80130 Naples, Italy; giuseppina.costabile@unina.it (G.C.); gdp0206@libero.it (G.D.P.); claudia.vetrani@unina.it (C.V.); ettoregri@gmail.com (E.G.); marilena.vitale@unina.it (M.V.); dominic_salamone91@libero.it (D.S.); annuzzi@unina.it (G.A.); lutgarda.bozzetto@unina.it (L.B.); 2 Department of Agricultural Sciences, Federico II University, 80055 Portici, Italy; paola.vitaglione@unina.it; 3 Institute of Food Sciences, National Research Council, 83100 Avellino, Italy; rgiacco@isa.cnr.it

**Keywords:** inflammation, C-reactive protein, interleukins, diet, polyphenols, n-3 fatty acids, fish

## Abstract

Interest has arisen on the anti-inflammatory action of dietary components, including long-chain n-3 fatty acids (LCn3) and polyphenols (PP). The aim of this study was to evaluate the effects of diets rich in PP and oily fish (high-LCn3 diets) on markers of subclinical inflammation and growth factors in people at high cardiometabolic risk. Individuals with high waist circumference and one more component of metabolic syndrome were randomized to one of the following isoenergetic diets: low LCn3&PP, high LCn3, high PP, high LCn3&PP. Before and after 8 weeks, fasting and postprandial plasma concentrations of hs-CRP and fasting serum concentrations of IL-1β, IL-4, IL-6, IL-10, IL-17, INF-γ, TNF-α, FGF, VEGF, PDGF-ββ, G-CSF, and GM-CSF were determined. An oily fish diet reduced fasting plasma hs-CRP (1.28 ± 12.0, −12.5 ± 6.9, 22.5 ± 33.6, −12.2 ± 11.9; 8-week percent change, Mean ± SEM; low LCn3&PP, high LCn3, high PP, high LCn3&PP group, respectively), postprandial 6h-AUC hs-CRP (4.6 ± 16.3, −18.2 ± 7.2, 26.9 ± 35.1, −11.5 ± 11.8, 8-week percent change) and fasting IL-6 (20.8 ± 18.7, −2.44 ± 12.4, 28.1 ± 17.4, −9.6 ± 10.2), IL-17 (2.40 ± 4.9, −13.3 ± 4.9, 3.8 ± 4.43, −11.5 ± 4.7), and VEGF (−5.7 ± 5.8, −5.6 ± 7.5, 3.5 ± 5.8, −11.1 ± 5.5) (8-week percent change; *p* < 0.05 for LCn3 effect for all; no significant effect for PP; 2-factor ANOVA). An oily fish diet improved subclinical inflammation, while no significant effect was observed for dietary polyphenols.

## 1. Introduction

Several studies have shown that subclinical inflammation plays an important role in numerous chronic diseases, such as cardiovascular disease (CVD), diabetes, obesity-related conditions, and autoimmune diseases [1]. Plasma C-reactive protein (CRP), an acute phase protein and marker of chronic low-grade inflammation, is associated with future cardiovascular risk in apparently healthy subjects [2,3] and is an independent risk factor for coronary heart disease (CHD) deaths in type 2 diabetes [4]. Moreover, other factors of the inflammatory cascade, including inflammatory cytokines (IL-1β, IL-6, IL-12, IL-17, TNF-α, G-CSF, and IFN-γ), anti-inflammatory cytokines (IL-4 and IL-10), and growth factors (VEGF, FGF, PDGF-ββ) are predictive for development of CVD [5] or cardiovascular events [6].

There is significant evidence that diet modulates the risk of CVD [7] by affecting low-grade inflammation status. The reduction in body weight is strongly and linearly associated with the reduction in CRP levels [8]. Beside diets to lose weight, isoenergetic dietary changes also may affect low-grade inflammation. To this respect, two dietary components, i.e., long-chain n-3 fatty acids (LCn3) and polyphenols, are considered as promising.

Several observational studies have suggested an anti-inflammatory effect of increased dietary intake or supplementation of LCn3 [9,10]. However, trials with LCn3 have generated contrasting results [11,12]. Most of the trials with marine LCn3, administered especially as fish oil supplements, have not shown any effect on circulating fasting inflammatory markers in healthy people [13,14], individuals with high risk of developing CVD, or individuals with type 2 diabetes [12], while decreased levels of circulating CRP and IL-6 have been observed in some other studies [15,16].

Few studies have evaluated the acute effect of LCn3 ingestion on the response of inflammatory markers in the postprandial state [17], which is a recognized inflammatory condition [18], while no study has investigated the effect of prolonged high LCn3 intake on postprandial inflammatory response.

Dietary polyphenols may have an important role in the prevention of CVD, diabetes, and neurodegenerative disorders [19,20]. In vitro studies have suggested that polyphenols may have anti-inflammatory effects through several mechanisms of action, including the downregulation of transcription factors [21], blocking the production of proinflammatory cytokines and the activation of anti-inflammatory cytokines [22]. The results of epidemiological and clinical intervention studies on the effects of polyphenol-rich foods on inflammation markers have been contradictory, reporting either neutral effects or variations in a single inflammatory biomarker or changes due to specific classes of polyphenols [23,24]. In general, the effects of polyphenols contained in individual foods (tea, red wine, cocoa, etc.) and not of diets rich in polyphenols from different sources have been investigated.

Therefore, the aim of the present study was to evaluate the medium-term effects of diets naturally rich in different sources of polyphenols and/or oily fish on the main markers of subclinical inflammation and growth factors in the fasting and postprandial conditions in a randomized controlled trial performed in people at high cardiovascular risk. This is part of a study for which the results on lipid and glucose metabolism have been already published [25,26].

## 2. Results

### 2.1. Participants’ Characteristics and Dietary Compliance

As previously reported [25], the compliance to the experimental diets was good in all groups and, as expected, the diets followed only differed for polyphenol, LCn3, and Vitamin D amounts Table 1).

At baseline, the four groups of participants did not differ significantly for age, anthropometrics, fasting, postprandial levels of hs-CRP, fasting levels of ILs, and growth factors (Table 2).

### 2.2. Fasting and Postprandial Plasma hs-CRP Concentrations

**Figure 1:**
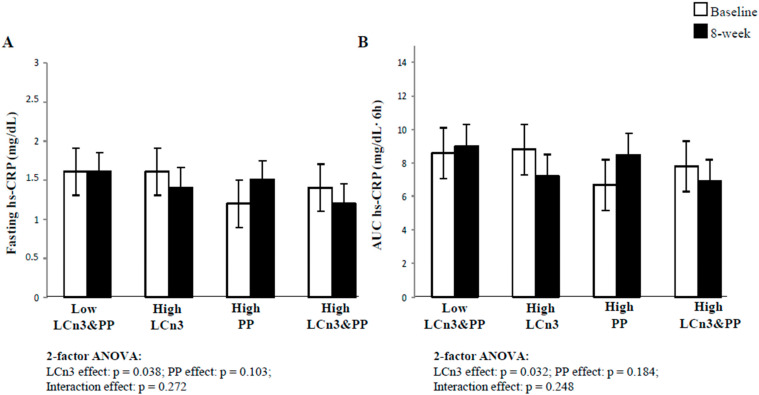
Fasting (**A**) and postprandial (**B**) plasma hs-CRP concentrations at baseline (white square) and after 8-week dietary intervention (black square) in the four experimental groups. Mean ± SEM. Comparisons made by 2-factors ANOVA of percent changes in plasma hs-CRP adjusted for sex, age, and baseline BMI. LCn3, long-chain n-3 fatty acids; PP, polyphenols. n = 14 for Control, n = 18 for High LCn3, n = 17 for High PP, n = 15 for High LCn3&PP group.

Baseline and 8-week plasma concentrations of fasting and postprandial hs-CRP are shown in Figure 1. Nine subjects (five in the Low LCn3&PP, one in the High PP group, and three in the High LCn3&PP group) were excluded from the analysis because their plasma hs-CRP levels were higher than 5 mg/dL, a value identified as marker of high-grade inflammation [27]. Fasting and postprandial hs-CRP levels were reduced by LCn3 intake (−12.5 ± 6.9 and −18.2 ± 7.2, mean ± SE, fasting and postprandial 8-week percent change, respectively) and increased by polyphenols (22.5 ± 33.6 and 26.9 ± 35.1, mean ± SE, fasting and postprandial 8-week percent change, respectively) with a significant effect for LCn3 by two-factor ANOVA, both at fasting (*p* = 0.038, Figure 1A) and postprandially (*p* = 0.032, Figure 1B).

### 2.3. Fasting Serum Ils and Growth Factors Concentrations

**Figure 2:**
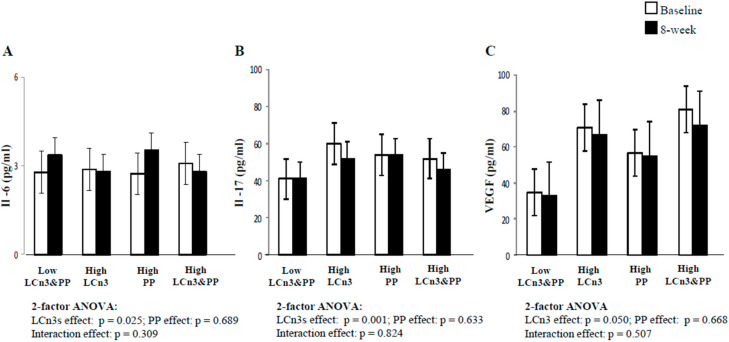
Fasting serum concentrations of IL-6 (**A**), IL-17 (**B**) and VEGF (**C**) at baseline (white square) and 8-week dietary intervention (black square) in the 4 experimental groups. Mean ± SEM. Comparisons made by 2 factor after ANOVA of percent changes in plasma hs-CRP adjusted for sex, age, and baseline BMI.LCn3, long-chain n-3 fatty acids; PP, polyphenols. For IL-6 (A): n = 12 for Control, n = 13 for High LCn3, n = 11 for High PP, n = 13 for High LCn3&PP group.

Baseline and 8-week fasting serum concentrations of IL-6, IL-17, and VEGF are shown in Figure 2. Subjects with undetectable levels of IL-6 (seven in the Low LCn3&PP, five in the High LCn3, seven in the High PP, and five in the High LCn3&PP group) were excluded from the analysis of IL-6. Fasting serum concentrations of IL-6 tended to increase at the end of the Low LCn3&PP and High PP diets (20.8 ± 18.7 and 28.1 ± 17.4, mean ± SE, fasting 8-week percent change, respectively), while there was no change and a small decrease after the High LCn3 and High LCn3&PP diets (−2.44 ± 12.4 and −9.6 ± 10.2, mean ± SE, fasting 8-week percent change, respectively), with a significant effect for LCn3 by two-factor ANOVA (*p* = 0.025) (Figure 2A). Fasting serum concentrations of IL-17 (Figure 2B) decreased after the two High LCn3 diets (−13.3 ± 4.9 and −11.5 ± 4.7, mean ± SE, fasting 8-week percent change, respectively) and were unchanged after the Low LCn3&PP and High PP diets (2.40 ± 4.9 and 3.80 ± 4.43, mean ± SE, fasting 8-week percent change, respectively), with a significant effect for LCn3 by 2-factor ANOVA (*p* = 0.001).

Fasting serum concentrations of VEGF (Figure 2C) decreased after the two High LCn3 diets (−5.6 ± 7.5 and −11.1 ± 5.5, mean ± SE, fasting 8-week percent change, respectively) and the Low LCn3&PP (−5.7 ± 5.8, mean ± SE, fasting 8-week percent change), and were unchanged after the High PP diets (3.5 ± 5.8, mean ± SE, fasting 8-week percent change, respectively), with a significant effect for LCn3 by two-factor ANOVA (*p* = 0.05).

No significant effects by LCn3, PP, or their interaction were observed for all other ILs and growth factors, as shown in Table 3 (*p* > 0.05 for all, two-factor ANOVA).

## 3. Discussion

The first relevant finding of this study is that oily fish-based diets naturally rich in LCn3 reduced plasma hs-CRP concentrations both at fasting and in the postprandial state and ameliorated the inflammatory status. An oily fish-based diet also reduced other components of the inflammatory cascade, particularly IL-6 and IL-17. Although the magnitude of the observed reduction was minimal, it is important to underline that it occurred in individuals with nonpathological levels of hs-CRP. Because of the growing evidence of the pathophysiological role of low-grade inflammation, these results obtained in a controlled randomized trial help define how oily fish intake may influence the risk of cardiometabolic diseases. Oily fish is an important source not only of LCn3 but also of vitamin D, which is known to have anti-inflammatory activity on Th17 cells to maintain immunological homeostasis [28].

In fact, in our study, in parallel with the increase in the intake of LCn3 with diets rich in oily fish, an increase in the intake of vitamin D was also observed. Therefore, we cannot exclude a possible role of vitamin D in the reduction of the inflammatory markers found in our study. The second finding of this study is that diets naturally rich in different polyphenols did not significantly affect inflammatory status. Previous evidence of the association between marine LCn3 and inflammation has mainly been found in cross-sectional epidemiological studies [9,10]. Fish consumption was independently associated with lower inflammatory markers in healthy adults in the ATTICA study in Greece [29], in an older Japanese population with a diet rich in marine products [10], and in the Nurses’ Health Study I cohort [23].

Intervention trials with marine LCn3 showed controversial results on circulating inflammatory markers, with most of the trials showing no effect [12,13] and others showing decreased levels of inflammatory markers [30]. Generally, those studies were conducted with fish-oil capsule supplementation and showed no significant effects on CRP levels in healthy individuals [31], healthy moderately obese individuals [32], dyslipidemic individuals with visceral obesity [33], or type 2 diabetic patients [12]. In contrast, a significant decrease in inflammatory markers in postmenopausal women on hormone replacement therapy was reported [34]. Results could differ with use of seafood. In fact, different effects between fish oil and whole fish intake were shown during an 8-week intervention trial, with greater reductions in hs-CRP levels observed in the salmon and cod groups than fish oil capsules or control (sunflower oil capsules, no seafood) groups, although weight loss was the only significant independent predictor of the reduction in hs-CRP [35]. Several meta-analyses have evaluated the effects of LCn3 on inflammatory status, again with controversial results [30,36]. In the meta-analysis by Li et al. [30], marine-derived LCn3 supplementation had a significant lowering effect on CRP. Subgroup analyses showed that the lowering effect of marine-derived LCn3 supplementation on CRP was more evident when placebo was linoleic acid and in studies with longer duration, higher daily dose of LCn3, older age, more females, and higher BMI. Marine-derived LCn3 from dietary intake showed a significant lowering effect on IL-6 but not on CRP and TNF-α. Different factors may explain the discrepancy, including the type of population studied, variations in dosage, treatment length, use of supplements or whole fish, confounding medications, different gender sensibility, and small sample size in many studies. In this randomized controlled trial, the improvement in inflammatory status was observed in an adequate sample of individuals at high cardiometabolic risk who were not on confounding pharmacologic treatments, with a feasible, accepted whole fish intake, at moderate doses of LCn3 (4 g/day) in the context of an isocaloric diet of sufficient duration.

In our study, we also observed a reduction in the AUC of hs-CRP after the meal rich in LCn3 with respect to the other test meals. This represents novel evidence of possible clinical relevance considering that the postprandial state is considered a well-known proinflammatory condition.

Several mechanisms shown in in vitro and animal studies have demonstrated the biological plausibility for LCn3 effects on systemic inflammation [37]. The possible interlinked mechanisms include modified cell membrane phospholipid fatty acid composition, disruption of lipid rafts, inhibition of activation of the proinflammatory transcription factor NFkB, activation of the anti-inflammatory transcription factor NR1C3 (i.e., PPAR-γ), and binding to the GPR120. LCn3 can inhibit the oxidation of arachidonic acid by cyclooxygenase (COX) enzymes, thus reducing the production of proinflammatory eicosanoids that play an important role in regulating the production of several cytokines, such as TNF-α and IL-6 [37]. In addition, LCn3 are known precursors of anti-inflammatory lipid mediators named protectins (from EPA) and resolvins (from EPA and DHA) [38].

In our intervention study, the diet rich in polyphenols had no effect on the inflammatory status. These results disagree with data from in vitro studies [21] and some, but not all, cross-sectional studies [23,39]. On the other hand, intervention studies in humans have shown conflicting results likely due to the type and source of polyphenols used in the different interventions [40,41,42]. Indeed, the chemical structure of polyphenols and their disposition in the food matrices, as well as the combined consumption with other foods, affect the pharmacokinetic parameters of the molecules and the antioxidant and ant inflammatory effects in the postprandial phase and in the long term [43].

This study presents some strengths and limitations. A strength is the good compliance to dietary treatment by the participants, which favored using foods included in a feasible and acceptable diet [25]. This was confirmed by the evaluation of urinary phenolic metabolites [20] and the incorporation of DHA and EPA in the HDL [44].

A limitation of our study is that the participants were at high cardiometabolic risk. Therefore, we do not know whether the same results would also apply to a “healthy” population or a population with a more advanced degree of metabolic or cardiovascular disease. In addition, the fewer data available for the IL-6 and postprandial hs-CRP values could represent another limitation of the study.

## 4. Materials and Methods

In total, 78 individuals of both genders aged 35–70 years, with overweight or obesity (BMI 27–35 kg/m^2^), high waist circumference (men > 102 cm, women > 88 cm), and at least 1 more criterion for metabolic syndrome diagnosis according to the NCEP/ATP III [27] were recruited at the obesity outpatient clinic of the Federico II University Hospital. Exclusion criteria were: Fasting plasma triglycerides ≥ 400 mg/dL, fasting cholesterol > 270 mg/dL, cardiovascular events (myocardial attack or stroke) during the 6 months prior to the study, established diabetes mellitus, intensive regular exercise activity, renal and liver failure or any other chronic disease, o use of drugs able to influence lipid or glucose metabolism and inflammation. The participants had stable food habits, were not vegetarians, and were asked to abstain from any dietary supplement for one month prior to and during the study. The design of the trial was approved by the Federico II University Ethics Committee, complied with the Helsinki Declaration guidelines, and was registered at ClinicalTrials.gov (accessed on 1 June 2021), number NCT01154478. All participants provided written informed consent. According to a 2 × 2 factorial design, participants were randomly assigned to 1 of 4 nutritional isoenergetic intervention arms for the duration of 8 weeks as previously described [25].

The assigned diets differed in LCn3 and polyphenols (PP) contents and were similar in the remaining macro- and micronutrient composition (Table 1). The 4 diets were: Low in LCn3 (1.5 g/day) and PP (365 mg/day); high in LCn3 (4 g/day) and low in PP (363 mg/day); high in PP (2903 mg/day) and low in LCn3 (1.4 g/day); and high in PP (2861 mg/day) and LCn3 (4 g/day). The difference in LCn3 and/or PP amount was obtained through the selection of specific foods and beverages. The main dietary sources of LCn3 were salmon (330 g twice a week), dentex, or anchovies (350 g once a week). Dietary PP were provided by daily intake of decaffeinated green tea (400 mL, 4 bags) and coffee (4 cups), dark chocolate (25 g), blueberry jam (40 g), extra-virgin olive oil (60 g), and polyphenol-rich vegetables (88 g rocket salad, 200 g fennels, 200 g onions). In the high-polyphenol diet, the main types of bioactive polyphenol compounds were anthocyanidins, flavones, flavonoids, phenolic acids, flavans, flavanones, and flavonols (Supplementary Table S1). Meals and beverages were provided to the participants for the whole study period in amounts sufficient to cover their household consumption. Meals were prepared in a qualified catering service under the surveillance of the dietitians. At baseline and after the 8-week intervention, body weight, height, and waist circumference were measured according to standardized procedures. After a 12-h overnight fast, the participants consumed a 1000 kcal test meal composed of rice, butter, parmesan cheese, bresaola, and white bread, with intakes of olive oil, extra-virgin olive oil, salmon, and decaffeinated green tea differing to obtain a similar composition as the assigned diet [25]. Blood samples were collected at fasting and 2 h, 4 h, and 6 h after the meal to measure high sensitivity CRP (hs-CRP), interleukins, and growth factors concentrations (only at fasting).

Plasma hs-CRP concentration was determined by a high-sensitivity immunoturbidimetric method (Roche Molecular Biochemicals, Mannheim, Germany) with a functional sensitivity of 0.11 mg/L. Intra- and inter-assay variability were, respectively, 0.3% and 1.93%. The Bio-Plex pro-human cytokine, chemokine, and growth factor assay kit (Bio-Rad Laboratories SRL, Segrate, Italy) allowed the simultaneous quantification of IL-1β, IL-4, IL-6, IL-10, IL-12, IL-17, TNF-α, interferon (IFN-γ), granulocyte colony-stimulating factor (G-CSF), vascular endothelial growth factor (VEGF), basic fibroblast growth factor (FGF), and platelet-derived growth factor subunit B (PDGF-ββ). The sensitivity levels of the assay (in pg/mL) correspond to the following: IL-1β, 0.24; IL-4, 0.09; IL-6, 0.34; IL-10, 0.69; IL-12, 0.78; IL-17, 1.16; TNF-α, 1.13; IFN-γ, 1.05; G-CSF, 3.63; VEGF, 10.16; FGF, 2.54; PDGF-ββ, 2.96. The inter-assay variation (% CV) was <10%, and the intra-assay variation (% CV) was <5%.

### Statistic Determinations

Data are expressed as mean ± SEM unless otherwise stated. Total postprandial areas under the curve (AUC) were calculated using trapezoidal rule. Measurements of the inflammatory markers were available for 73 subjects who were therefore included in the analysis (Table 2). The differences in baseline characteristics between the 4 groups were analyzed by ANOVA and Least Significant Difference (LSD) post-hoc analysis. According to the 2 × 2 factorial design, the effects of dietary PP and LCn3 and their interaction were evaluated by 2-factor ANOVA analysis. In the General Linear Model (GLM)-Univariate Analysis, percent changes of hs-CRP, interleukins, and growth factors concentrations (∆% = 8 weeks minus baseline/baseline × 100) was added as a “dependent variable.” The PP group (with 2 levels: Low PP and high PP) and LCn3 group (with 2 levels: Low LCn3 and high LCn3) were added as “independent variables/fixed factors,” and sex, age, and baseline BMI were added as covariates.

For all analyses, the level of statistical significance was set at *p* < 0.05 (2 tails). Statistical analysis was performed according to standard methods using the Statistical Package for Social Sciences software version 21.0 (SPSS, Chicago, IL, USA).

## 5. Conclusions

In conclusion, this randomized controlled trial showed that a diet enriched in polyphenols from different food sources did not modify individual inflammatory status, whereas a diet enriched in LCn3 from oily fish improved inflammation in individuals at high risk of diabetes and cardiovascular disease. This effect may contribute to the overall favorable effects of fish consumption in the prevention and therapy of cardiovascular disease.

## Figures and Tables

**Table 1:** Mean composition of the diets assigned per protocol and the diets followed in the four groups of participants in the dietary intervention study.

	Low LCn3&PP (n = 20)		High LCn3 (n = 19)		High PP (n = 20)		High LCn3&PP (n = 19)	
	Assigned ^1^	Followed ^2^	Assigned ^1^	Followed ^2^	Assigned ^1^	Followed ^2^	Assigned ^1^	Followed ^2^
Total Energy (kcal)	2524 ± 366	2345 ± 472	2718 ± 283	2602 ± 248	2622 ± 456	2539 ± 459	2507 ± 452	2407 ± 471
Proteins (E%)	15.7 ± 0.0	16.0 ± 1.2	15.7 ± 0.0	15.9 ± 0.6	15.7 ± 0.0	15.8 ± 0.5	15.7 ± 0.0	15.8 ± 0.6
Total fat (E%)	33.6 ± 0.0	32.5 ± 1.7	33.6 ± 0.0	33.2 ± 1.9	33.6 ± 0-0	33.9 ± 1.4	33.6 ± 0.0	33.8 ± 1.5
Saturated fat (E%)	7.2 ± 0.1	7.2 ± 0.3	7.3 ± 0.0	7.3 ± 0.4	7.1 ± 0.0	7.3 ± 0.4	7.2 ± 0.3	7.4 ± 0.5
MUFA (E%)	21.0 ± 0.2	20.0 ± 1.6	19.9 ± 0.2	19.4 ± 1.5	19.2 ± 0.3	19.3 ± 1.1	18.6 ± 0.4	18.8 ± 1.0
PUFA n-6 (E%)	3.1 ± 0.1	3.1 ± 0.2	2.7 ± 0.0	2.7 ± 0.2	2.8 ± 0.2	2.9 ± 0.3	2.7 ± 0.1	2.7 ± 0.2
PUFA n-3 (E%)	0.5 ± 0.0	0.5 ± 0.0	1.5 ± 0.0	1.4 ± 0.1 *	0.5 ± 0.4	0.5 ± 0.1	1.5 ± 0.1	1.5 ± 0.2 *
EPA (E%)	0.02 ± 0.01	0.02 ± 0.01	0.43 ± 0.02	0.40 ± 0.06 *	0.04 ± 0.01	0.04 ± 0.01	0.47 ± 0.02	0.46 ± 0.06 *
DHA (E%)	0.01 ± 0.01	0.01 ± 0.01	0.58 ± 0.02	0.53 ± 0.08 *	0.01 ± 0.0	0.01 ± 0.01	0.60 ± 0.03	0.59 ± 0.08 *
Cholesterol (mg)	191 ± 3	184 ± 27	195 ± 1	192 ± 32	178 ± 6	187 ± 16	198 ± 8	197 ± 24
Total CHO (E%)	50.7 ± 0.0	51.5 ± 1.0	50.7 ± 0.0	50.9 ± 1.9	50.7 ± 0.0	50.4 ± 1.4	50.7 ± 0.0	50.3 ± 1.9
Fiber (g)	28.7 ± 0.2	26.8 ± 4.4	28.4 ± 0.2	27.8 ± 3.9	29.0 ± 0.9	27.5 ± 2.2	28.4 ± 0.3	27.2 ± 2.8
Vitamin C (mg)	279 ± 0.0	261 ± 24	284 ± 0.0	256 ± 38	279 ± 0.0	256 ± 21	275 ± 0.0	257 ± 24
Vitamin E (mg)	17.3 ± 2.0	15.8 ± 3.2	18.1 ± 1.5	17.5 ± 2.2	18.7 ± 2.5	18.0 ± 2.7	18.6 ± 3.0	17.7 ± 3.0
Vitamin D (μg)	2.54 ± 0.20	1.83 ± 0.71	9.96 ± 1.68	13.98 ± 1.81 *	2.36 ± 0.46	2.01 ± 0.64	10.82 ± 1.93	10.26 ± 2.17 *
Polyphenols (mg)	365 ± 3	336 ± 79	363 ± 2	377 ± 55	2903 ± 19	2776 ± 234 **^§^**	2861 ± 42	2667 ± 400 **^§^**

All values are means ± SDs. Comparisons between Assigned and Followed diets were not statistically significant (*t*-test, *p* value > 0.05 for all dietary components). * Significantly different from the Low LCn3&PP and High PP groups, *p* < 0.0001 (ANOVA and least significant difference post-hoc test). ^§^ Significantly different from the Low LCn3&PP and High LCn3 groups, *p* < 0.0001 (ANOVA and least-significant-difference post-hoc test). LCn3, long-chain n-3 polyunsaturated fatty acids; PP: Polyphenols; E%, percent of total energy intake; MUFA, monounsaturated fatty acids; PUFA, polyunsaturated fatty acids; EPA, eicosapentaenoic acid; DHA, docosahexaenoic acid; CHO, carbohydrate. ^1^ Data for Assigned diets include the changes made in different visits during the trial to keep body weight stable. ^2^ Data for Followed diets were calculated from the 7-day food records at weeks 4 and 8.

**Table 2:** Baseline characteristics and inflammatory markers concentrations of the four groups of participants in the dietary intervention study.

	Low LCn3&PP (n = 19)	High LCn3 (n = 18)	High PP (n = 18)	High LCn3&PP (n = 18)	*p* Value (ANOVA)
Gender (M/F)	7/12	8/10	8/10	7/11	-
Age (years)	54 ± 2	56 ± 2	52 ± 2	54 ± 2	0.658
BMI (kg/m^2^)	33 ± 1	31 ± 1	32 ± 1	30 ± 1	0.090
Plasma hs-CRP (mg/dL) ^1^	1.6 ± 0.4	1.6 ± 0.3	1.2 ± 0.3	1.3 ± 0.3	0.700
Plasma AUC hs-CRP (mg/dL·6 h) ^1^	8.6 ± 2.0	8.8 ± 1.1	6.7 ± 1.0	7.8 ± 1.1	0.759
Serum IL-1 β (pg/mL)	0.12 ± 0.03	0.05 ± 0.02	0.07 ± 0.03	0.18 ± 0.11	0.486
Serum IL-4 (pg/mL)	4.0 ± 0.7	4.6 ± 0.6	4.7 ± 0.8	4.6 ± 0.6	0.850
Serum IL-6 (pg/mL) ^2^	2.8 ± 0.7	2.9 ± 0.5	2.7 ± 0.4	3.1 ± 0.5	0.787
Serum IL-10 (pg/mL)	3.3 ± 1.7	4.2 ± 1.0	2.0 ± 0.6	7.9 ± 3.5	0.200
Serum IL-12 (pg/mL)	5.8 ± 1.3	13.2 ± 3.7	8.3 ± 2.3	17.4 ± 5.8	0.116
Serum IL-17 (pg/mL)	41.5 ± 8.9	59.7 ± 10.8	53.9 ± 10.8	52.5 ± 8.4	0.607
Serum TNF-α (pg/mL)	2.6 ± 0.7	3.7 ± 0.8	3.8 ± 0.8	3.5 ± 0.7	0.702
Serum IFN-γ (pg/mL)	71.3 ± 10.7	79.7 ± 10.2	63.6 ± 9.5	68.4 ± 8.4	0.708
Serum G-CSF (pg/mL)	2.4 ± 0.5	3.6 ± 0.7	2.5 ± 0.6	3.0 ± 0.6	0.512
Serum PDGF-ββ (pg/mL)	2880 ± 700	2882 ± 635	3153 ± 698	3044 ± 545	0.988
Serum FGF (pg/mL)	23.8 ± 5.1	41.9 ± 8.2	26.8 ± 6.8	32.3 ± 6.2	0.244
Serum VEGF (pg/mL)	34.9 ± 7.3	71.2 ± 17.3	56.7 ± 12.5	80.8 ± 22.7	0.188

Data are expressed as Mean ± SEM. AUC = area under the curve; BMI = body mass index; IL = interleukins; TNF = tumor necrosis factor; IFN = interferon; G-CSF = Granulocyte colony-stimulating factor; PDGF = platelet-derived growth factor; FGF = fibroblast growth factor; VEGF = vascular endothelial growth factor. LCn3 = long-chain n3 fatty acids; PP = polyphenols. ^1^ n = 14 for Low LCn3&PP, n = 18 for High LCn3, n = 17 for High PP, n = 15 for High LCn3&PP group. ^2^ n = 12 for Low LCn3&PP, n = 13 for High LCn3, n = 11 for High PP, n = 13 for High LCn3&PP group.

**Table 3:** Fasting serum concentrations of interleukins and growth factors before (Baseline) and after 8-week intervention.

	Low LCn3&PP (n = 19)		High LCn3 (n = 18)		High PP (n = 18)		High LCn3&PP (n = 18)	
	Baseline	End	Baseline	End	Baseline	End	Baseline	End
IL1-β (pg/mL)	0.12 ± 0.03	0.14 ± 0.03	0.05 ± 0.02	0.05 ± 0.02	0.07 ± 0.02	0.08 ± 0.02	0.18 ± 0.11	0.07 ± 0.03
IL-4(pg/mL)	4.0 ± 0.7	3.9 ± 0.6	4.6 ± 0.6	4.5 ± 0.7	4.7 ± 0.8	4.8 ± 0.8	4.6 ± 0.6	4.6 ± 2.8
IL-10 (pg/mL)	3.3 ± 1.7	3.3 ± 1.8	4.2 ± 1.0	2.7 ± 0.6	2.0 ± 0.6	1.9 ± 0.6	7.9 ± 3.5	5.3 ± 2.1
IL-12 (pg/mL)	5.8 ± 1.3	4.8 ± 0.9	13.2 ± 3.7	10.0 ± 3.0	8.3 ± 2.3	8.3 ± 2.4	17.4 ± 5.8	15.0 ± 4.9
IFN-γ (pg/mL)	71.3 ± 10.7	72.7 ± 12.0	79.7 ± 10.2	73.7 ± 9.8	63.6 ± 9.5	67.6 ± 11.3	68.4 ± 8.4	70.9 ± 9.9
PDGF-ββ (pg/mL)	2880 ± 700	2633 ± 589	2882 ± 635	2874 ± 623	3153 ± 698	3204 ± 657	3044 ± 545	2962 ± 597
TNF-α (pg/mL)	2.6 ± 0.7	2.5 ± 0.7	3.7 ± 0.8	3.5 ± 0.8	3.8 ± 0.8	3.24 ± 0.79	3.5 ± 0.7	3.3 ± 0.7
FGF basic (pg/mL)	23.8 ± 5.1	21.1 ± 3.8	42.0 ± 8.2	37.0 ± 8.3	26.8 ± 6.8	28.4 ± 7.8	32.3 ± 6.2	25.6 ± 4.6
G-CSF (pg/mL)	2.4 ± 0.5	2.4 ± 0.4	3.6 ± 0.7	3.1 ± 0.7	2.5 ± 0.6	2.7 ± 0.6	3.0 ± 0.6	2.6 ± 0.5

Data are expressed as Mean ± SEM. IL = interleukins; TNF = tumor necrosis factor; IFN = interferon; G-CSF = Granulocyte colony-stimulating factor; PDGF = platelet-derived growth factor; FGF = fibroblast growth factor; LCn3 = long-chain n-3 fatty acids; PP = polyphenols.

## Data Availability

Not applicable.

## Supplementary Materials

The following are available online, Table S1: Mean composition of the diets assigned per protocol and the diets followed in the four groups of participants in the dietary intervention study.

## Sample Availability

Samples of the compounds are available from the authors.
