# Supplementary material for: An Oily Fish Diet Improves Subclinical Inflammation in People at High Cardiovascular Risk: A Randomized Controlled Study
Source: Molecules. 2021 Jun 2;26(11):3369. doi: 10.3390/molecules26113369 (PMC8199776; doi:10.3390/molecules26113369)
Supplement: Supplementary file 1 [file molecules-26-03369-s001.zip › molecules-1214617-supplementary.pdf]

Supplementary Table 1. Absolute changes[a] from baseline of the mean daily intakes (mg/day) of total dietary polyphenols and their classes in the study groups

	High-polyphenol diets (n = 39)		Low-polyphenol diets (n = 39)	
	mg/day	%*	mg/day	%*
Total polyphenols	2025 ± 574	+ 305	− 239 ± 294	− 39
Phenolic acids	628 ± 322	+ 125	− 216 ± 300	− 40
Total flavonoids	1360 ± 303	+ 1782	− 22.6 ± 39	− 35
Anthocyanidins	78.2 ± 47	+ 844	13.0 ± 15	+ 124
Flavones	4.50 ± 2.5	+ 227	0.60 ± 1.7	+ 33
Flavan-3-ols	1007 ± 266	+ 4698	− 20.5 ± 16	− 99
Flavonols	208 ± 78	+ 990	− 4.5 ± 7.9	− 39
Flavanones	62.1 ± 46	+ 277	− 11.2 ± 28	− 51

Data are expressed as mean ± SD

*Percentage increase/decrease from baseline calculates as: [(8-week value minus baseline value)/baseline value] × 100

[a] Calculated as 8-week value minus baseline value
